# Radiation Safety Knowledge, Perceptions, and Self-Reported Practices Among Healthcare Workers in Radiology and Nuclear Medicine: A Cross-Sectional Survey

**DOI:** 10.3390/healthcare14152370

**Published:** 2026-08-03

**Authors:** Eda Kaya Pepele, Serdar Deniz

**Affiliations:** 1 Biomedical Devices Technology Program, Department of Electronics and Automation, Yeşilyurt Vocational School of Technical Sciences, Malatya Turgut Ozal University, 44900 Malatya, Turkey; 2 Department of Public Health, Faculty of Medicine, Malatya Turgut Özal University, 44900 Malatya, Turkey; serdar.deniz@ozal.edu.tr

**Keywords:** radiation safety, radiation protection, healthcare workers, radiology, nuclear medicine, occupational exposure, dosimetry, personal protective equipment

## Abstract

**Highlights:**

**What are the main findings?**

- More than half of healthcare workers (61.6%) reported not using any personal protective equipment in routine practice, and 21.0% reported not using a dosimeter despite working in radiation-exposed units.
- Knowledge of the 5-year average occupational dose limit was markedly limited, with only 13.8% of participants answering correctly.

**What are the implications of the main findings?**

- The findings identify potential targets for future quality-improvement efforts, including role-specific education and institutional radiation safety processes.

**Abstract:**

**Background/Objectives**: This study aimed to evaluate radiation safety knowledge, perceptions, and self-reported protective practices among healthcare workers (HCWs) working in radiology and nuclear medicine departments, with particular attention to occupational dose limits, radiation protection principles, dosimetry, and personal protective equipment use. **Methods**: A cross-sectional survey was conducted among 138 HCWs (25 specialist doctors, 101 technicians, 9 nurses and 3 radiophysicists) in radiology and nuclear medicine departments from May to June 2024. Data were collected using a structured 31-item survey assessing sociodemographic characteristics, perceived radiation exposure risk, knowledge of radiation protection principles and occupational dose limits, dosimeter use, personal protective equipment use, and awareness of institutional radiation safety procedures. The total knowledge score was calculated by summing correct responses to knowledge items (range: 0–10) and analyzed as a continuous outcome using multivariable linear regression with HC3 robust standard error. Because of the small and uneven distribution of some subgroups, item-level subgroup comparisons were interpreted descriptively and cautiously. **Results**: Although most HCWs reported receiving radiation safety information through formal education or institutional training, important knowledge and self-reported practice gaps remained. While 96.4% perceived themselves to be at high risk of radiation exposure, 61.6% reported not using any PPE in routine practice and 21.0% reported not using a dosimeter. Only 13.8% correctly identified the 5-year average permissible occupational dose, whereas 32.6% correctly identified the annual maximum dose limit. Knowledge of ionizing-radiation methods was higher among postgraduates than undergraduates (100% vs. 27.5%, *p* < 0.001) and among nuclear medicine staff than radiology staff (100% vs. 37.8%, *p* < 0.001). Knowledge of all three basic radiation protection methods was also higher among postgraduates than undergraduates (100% vs. 15.6%, *p* < 0.001). Because some subgroup distributions were small and uneven, these item-level subgroup findings should be interpreted descriptively and with appropriate caution. In multivariable linear regression with HC3 robust standard errors, technicians (B = −3.09, 95% CI = −3.52 to −2.67, *p* < 0.001) and nurses (B = −3.84, 95% CI = −5.26 to −2.43, *p* < 0.001) had lower total knowledge scores than specialist doctors. Nuclear medicine was associated with a higher total score than radiology (B = 2.33, 95% CI = 0.81 to 3.85, *p* = 0.003), whereas >3 years of work experience was associated with a lower score than ≤3 years (B = −1.53, 95% CI = −2.40 to −0.66, *p* = 0.001). When postgraduate education was evaluated instead of professional role because of collinearity, postgraduate education was associated with a higher total knowledge score. Because of the cross-sectional design, these associations should not be interpreted as causal. The internal consistency of the knowledge subscale was minimally acceptable (Cronbach’s alpha = 0.694). **Conclusions**: Significant gaps and variations were observed in radiation safety knowledge and self-reported practices among HCWs. These patterns differed according to educational level, professional role, departmental affiliation, and experience; however, they should be interpreted in light of the cross-sectional design and the small and uneven distribution of some subgroups. These findings identify potential areas for future institutional quality-improvement efforts, including targeted education programs and review of radiation safety policies, whose effectiveness should be evaluated in future studies.

## 1. Introduction

Ionizing radiation underpins many core diagnostic and therapeutic practices in contemporary healthcare, with radiology and nuclear medicine representing the main clinical domains of its use. Worldwide, an estimated 4.2 billion diagnostic radiology examinations, approximately 40 million nuclear medicine procedures, and approximately 8.5 million radiotherapy treatments are performed annually [1]. As the volume of these procedures increases, the cumulative occupational exposure of healthcare workers (HCWs) in these environments also increases. Occupational radiation exposure is associated with deterministic effects at sufficient doses (e.g., skin injury and bone marrow suppression) and stochastic outcomes whose probability rises with exposure (e.g., malignancy, cataracts, and potential fertility impairment) [2,3,4]. Evidence derived from pooled investigations of heterogeneous cohorts of HCWs occupationally exposed to low-dose ionizing radiation, including personnel engaged in radiology, nuclear medicine, radiotherapy, and laboratory environments, shows elevated levels of DNA damage biomarkers among exposed individuals relative to unexposed control populations, thereby suggesting a potential long-term carcinogenic risk [5]. In parallel, epidemiological evidence obtained from heterogeneous cohorts of medical radiation workers indicates that cancer risk may be heightened within select subgroups characterized by comparatively greater exposure levels, most notably those engaged in fluoroscopically guided interventional procedures and radionuclide or nuclear medicine practices [6,7,8]. These findings emphasize the need for effective radiation safety protocols to protect HCWs from occupational exposure.

International guidelines, including International Commission on Radiological Protection (ICRP) occupational limit recommendations, set the average allowable dose at 20 mSv per year over a five-year period, with no single year exceeding 50 mSv, highlighting the “as low as reasonably achievable” (ALARA) principle [9,10]. For practice, radiation protection relies on the time-, distance-, and shielding-based design principle: minimize exposure time and increase distance from the source. In addition, personal protective equipment is applied, such as lead aprons (≥0.5 mm lead equivalence), thyroid shields, leaded eyewear, and protective gloves [11]. Frequent use of personal dosimetry and structural protective barriers is important to prevent cumulative exposures in procedures with higher scatter levels [12]. In view of this background, knowledge of occupational dose limits and the ALARA principle is essential in daily clinical practice because it enables HCWs to interpret monitored doses, comply with local radiation protection rules, use dosimeters and protective equipment correctly, and optimize exposure during routine and higher-exposure procedures [13].

Despite these measures, studies conducted in different healthcare systems reveal persistent gaps in awareness and protective practices. A systematic review encompassing 41 studies and 11,050 HCWs across several countries demonstrated that in the majority of reported cases, more than half of the HCWs interviewed possessed only modest knowledge of radiation protection principles and exhibited suboptimal protective practices [14]. In a Jordanian hospital survey drawing HCWs from five separate institutions, mean scores indicated approximately 52% proficiency in knowledge of radiation protection, with approximately 37% awareness of radiation hazards [15]. This study further reported that participants’ professional experience is primarily associated with a weak level of understanding of radiation protection principles and related hazards [15]. At a large tertiary medical center in Israel, a comparative assessment conducted among HCWs encompassing physicians, nurses, allied health professionals, and ancillary and administrative staff revealed that physicians demonstrated superior levels of knowledge and awareness regarding radiation hazards relative to their counterparts in other occupational categories. An identical pattern was observed in workers assigned to workplace areas where exposure to radiation was highly likely [16]. In a study carried out in Egypt among HCWs occupationally exposed to ionizing radiation, nearly two-thirds of the participants held the false belief that magnetic resonance imaging (MRI) and ultrasound utilize X-rays, while nearly half reported not employing dosimetric monitoring devices nor wearing lead collars [17]. Consistently with these observations, other studies conducted in Hong Kong and Spain have also documented that many physicians underestimate the radiation doses associated with common imaging exams and are unaware of the cumulative risks [18,19]. Conversely, studies involving HCWs in public tertiary care hospitals in Trinidad and neonatal and pediatric intensive care nurses in public tertiary and pediatric hospitals in Türkiye show that targeted educational interventions can improve knowledge and support safer protective practices. HCWs who have attended formal radiation safety courses or in-service training sessions tend to score higher on radiation protection knowledge and demonstrate better adherence to safety protocols [20,21].

Given the occupational risks associated with ionizing radiation, it is important to evaluate HCWs’ radiation safety knowledge, risk perceptions, and self-reported protective practices in routine clinical settings. This is particularly relevant in departments with regular but different patterns of radiation exposure. Accordingly, the present study focused specifically on radiology and nuclear medicine staff, as both groups encounter ionizing radiation in routine clinical workflows, but differ in exposure pathways, operational procedures, and radiation safety requirements. Radiology is principally concerned with X-ray-based diagnostic imaging and fluoroscopically guided interventional procedures, whereas nuclear medicine additionally encompasses the handling of radiopharmaceutical agents and the implementation of contamination-control measures. International guidance recognizes radiology and nuclear medicine as related, but distinct areas of medical radiation use, and multicenter surveillance data have reported differences in occupational exposure patterns among hospital-based radiation workers, including nuclear medicine personnel [22,23]. We hypothesized that radiation safety knowledge and self-reported protective practices would differ according to education level, professional role, department, and years of work experience, with higher knowledge expected among staff with higher educational attainment and among personnel working in more specialized radiation safety workflows, such as nuclear medicine. These factors were evaluated as associations within a cross-sectional survey design. Accordingly, this study aimed to evaluate the knowledge, perceptions, and self-reported practices of HCWs, including specialist doctors, technicians, nurses, and radiophysicists working in the departments of radiology and nuclear medicine regarding radiation health and safety, occupational dose limits, self-protection from radiation, and the use of radiation protection tools.

## 2. Materials and Methods

This cross-sectional, questionnaire-based study was carried out in the radiology and nuclear medicine departments of Malatya Training and Research Hospital, a tertiary public hospital, from May to June 2024. All procedures adhered to the ethical standards outlined by the Institutional Research Committee and were aligned with the 1964 Helsinki Declaration and its subsequent amendments. This study was approved by the Ethics Committee of Malatya Training and Research Hospital (16 April 2024, 220543). Written informed consent was obtained from all participants.

The sample size calculation was performed using a conservative single-proportion framework for the planned survey assessment of radiation safety knowledge among HCWs. This binary planning proportion was derived from the total knowledge score (range: 0–10) using a median split. Because no reliable prior estimate was available for this proportion in our institutional setting, an expected frequency of 50% was used. This is the most conservative assumption for a single-proportion sample size calculation, as it maximizes the *p* × (1 − *p*) term and therefore yields the largest required sample size for a given confidence level and margin of error. Accordingly, the minimum required sample size was calculated as 112 participants using Epi Info, version 7.2.6.0 (Centers for Disease Control and Prevention, Atlanta, GA, USA) assuming a 95% confidence level and a 5% margin of error. This calculation was intended to support the overall survey estimate and was not designed as a formal power calculation for subgroup comparisons or multivariable regression models.

### 2.1. Study Population

During the May–June 2024 study period, a total of 159 healthcare workers were employed in the radiology and nuclear medicine departments across all routine working shifts. Of these, 138 HCWs (86.8%) agreed to participate in the survey and completed the questionnaire. Because individual-level data were not available for non-participants, participants and non-participants could not be formally compared. The participating staff included healthcare workers from all routine working shifts, and the study period corresponded to routine departmental functioning without any extraordinary service interruption or temporary workflow change. The professional distribution of participants was as follows: specialist doctors (*n* = 25), technicians (*n* = 101), nurses (*n* = 9), and radiophysicists (*n* = 3).

**Figure 1:**
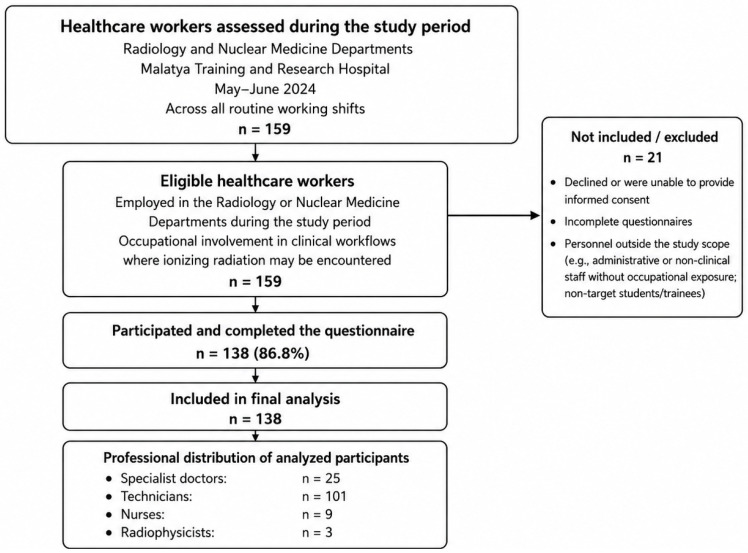
Participant flow diagram.

Inclusion criteria were employment in the radiology or nuclear medicine departments during the study period, occupational involvement in clinical workflows where ionizing radiation may be encountered, and voluntary agreement to participate. Exclusion criteria included refusal or inability to provide informed consent, incomplete questionnaires, and personnel outside the study scope (e.g., administrative/non-clinical staff without occupational exposure and students/trainees if not targeted) (Figure 1).

### 2.2. Study Protocol

All eligible healthcare professionals employed within the radiology and nuclear medicine departments during the designated study period were invited to participate in the study. To minimize interviewer bias, all face-to-face interviews were conducted by a single researcher using a predetermined standard question flow and a brief standardized explanatory script.

Data were collected through face-to-face interviews using a structured 31-item questionnaire assessing radiation safety knowledge, awareness, perceptions, and self-reported practices.

The questionnaire included a sociodemographic section (age, sex, education, unit, and professional experience) and items related to radiation exposure dose limits (permitted average and maximum dose limits) and radiation health and safety practices (e.g., dosimeter use, PPE use, radiation applications, and awareness of the radiation safety committee). All ionizing-radiation procedures—including chest X-ray, mammography, PET, fluoroscopy, interventional radiology, cardiac angiography, radiotherapy, bone densitometry, CT, scintigraphy, and I-131 therapy—were considered. In this framework, knowledge referred to items scored against predefined correct answers, including ionizing-radiation modalities, radiation protection principles, occupational dose limits, dosimeter placement, radiation worker health-form requirements, and radiation safety procedures for women of childbearing age. Awareness referred to recognition of the radiation safety committee, its responsibilities, radiation safety training, and radiation safety procedure documents. Perceptions included self-rated knowledge, perceived radiation exposure risk, and perceived effectiveness of training and regulations. Self-reported practices referred to reported PPE and dosimeter use, reasons for not using a dosimeter, measures taken during high exposure, and informing patients about radiation risks. All data were cross-checked against the original records, and a data audit was conducted prior to analysis. Item-level respondent counts (*n*) and denominators (N = 138, unless otherwise stated) were verified for consistency.

The questionnaire was developed based on relevant radiation protection guidance and adapted from previously published hospital-based surveys rather than a single fully psychometrically validated scale. Although the questionnaire was not a fully psychometrically validated scale, its content was informed by radiation protection guidance, previous hospital-based surveys, expert review, and pilot testing. Content clarity and relevance were reviewed by domain experts in radiation safety/medical physics. To ensure the clarity and comprehensibility of the questionnaire items, a pilot test was conducted with 15 HCWs who were not included in the main study, and minor wording revisions were made accordingly. The internal consistency of the knowledge-based items was assessed using Cronbach’s alpha, which indicated minimally acceptable consistency (Cronbach’s alpha = 0.694).

For knowledge items, responses were coded as correct or non-correct according to ICRP-based occupational dose limits and institutional radiation safety rules, and a total knowledge score was computed by summing correct responses (range: 0–10). No partial credit was assigned. This all-or-none scoring approach was used to preserve strict predefined correctness for safety-critical knowledge items and to avoid classifying incomplete or conceptually mixed responses as fully correct; however, it may have underestimated partial knowledge. For multi-component items (e.g., the three basic methods of radiation protection: time, distance, and shielding), responses were considered correct only when all required components were identified. For multi-response items assessing ionizing-radiation modalities, responses were classified as correct only if participants selected appropriate modalities without marking non-ionizing options, such as MRI or ultrasound. For single-response knowledge items, correct answers were predefined as follows: the ALARA principle as the lowest possible radiation dose; the 5-year average occupational dose limit as 20 mSv; the annual maximum occupational dose limit as 50 mSv; correct dosimeter placement as under the lead apron; radiation worker health form completion as once every 6 months; pregnancy questioning before examinations using ionizing radiation in women of childbearing age as “Yes,” appropriate management of suspected pregnancy as referral to the requesting physician; and the safest time for ionizing-radiation procedures in women of childbearing age as during menstruation and the following 10 days.

Items based primarily on international or national radiation protection guidance included the ALARA principle as the lowest reasonably achievable radiation dose, the 5-year average occupational dose limit as 20 mSv, the annual maximum occupational dose limit as 50 mSv, identification of ionizing-radiation modalities, the time–distance–shielding principle, and general dosimeter monitoring principles [9,22,24]. Items based on national or local procedural rules and institutional procedures included radiation worker health surveillance documentation, pregnancy questioning before examinations using ionizing radiation in women of childbearing age, appropriate management of suspected pregnancy through referral to the requesting physician, and the safest timing for ionizing-radiation procedures in women of childbearing age as applied in the institutional questionnaire [24,25].

### 2.3. Statistical Analysis

Statistical analyses were performed using SPSS software (version 26; IBM Corp., Armonk, NY, USA). The Shapiro–Wilk test was used to assess normality. Descriptive statistics are presented as means ± standard deviation for normally distributed continuous variables, median (25th–75th percentile) for non-normally distributed continuous variables, and frequency (percentage) for categorical variables.

Between-group comparisons of continuous variables were performed using one-way ANOVA with Bonferroni post hoc tests for normally distributed variables or the Kruskal–Wallis H test with Dunn’s post hoc test for non-normally distributed variables. Between-group comparisons of categorical variables were performed using the chi-squared test or Fisher’s exact test as appropriate. For categorical subgroup comparisons, Cramer’s V was calculated to describe the magnitude of association. Where post hoc pairwise comparisons were performed, Bonferroni adjustment was applied as appropriate.

The primary analytic outcome of the study was the total knowledge score, treated as a continuous scale (range: 0–10) to avoid information loss from dichotomization and arbitrary threshold selection. Because the knowledge score was bounded and the model residuals showed deviations from normality and heteroskedasticity, multivariable linear regression was performed using HC3 heteroskedasticity-consistent robust standard errors to obtain more robust statistical inference [26,27]. The following covariates were selected a priori based on clinical relevance and study design: sex, education level, professional role, department, and years of experience. Because education level and professional role were structurally related and produced problematic multicollinearity when entered simultaneously, two separate multivariable models were constructed. Model 1 included sex, education level, department, and years of experience. Model 2 included sex, professional role, department, and years of experience. Professional role was entered using specialist doctors as the reference category. Radiophysicists were excluded from regression modeling because of the very small subgroup and are presented descriptively.

Unstandardized regression coefficients (B) with HC3 robust standard errors and 95% confidence intervals (CIs) are reported. Multicollinearity among independent variables was assessed using variance inflation factors (VIFs) and tolerance values from the corresponding ordinary least squares models using the same covariate sets. A VIF value > 5 and a tolerance value < 0.2 were considered indicative of problematic collinearity.

Item-level knowledge and self-reported practice outcomes were analyzed as exploratory subgroup comparisons. These item-level outcomes were not modeled as separate multivariable outcomes because several subgroup combinations showed sparse or uneven distributions, with evidence of quasi-complete separation in some instances. Therefore, item-level findings should be interpreted as descriptive and exploratory rather than as evidence of independent group effects. Given the number of item-level subgroup comparisons, statistically significant findings were interpreted cautiously and in conjunction with effect size estimates.

All statistical tests were two-sided, and a *p*-value of <0.05 was considered statistically significant.

## 3. Results

The participants had a mean age of 39.4 ± 8.2 years, and the majority were female (57.2%). According to education level, 79.0% of the participants held a bachelor’s degree, while 18.1% were specialist doctors. Overall, 92% worked in the radiology unit, and 84.1% had more than three years’ experience in radiation fields (Table 1).

All respondents stated that they had at least some knowledge of radiation protection, most commonly acquired through structured training programs (94.9%) and institutional in-service education (62.3%). Among participants, 96.4% perceived their unit as having a higher radiation exposure risk than other clinical departments. Despite this perceived risk, 61.6% reported not using any of the listed PPE items in routine practice: in the questionnaire, the “None” option was mutually exclusive. Regarding personal dosimetry, 75.4% reported using only pocket dosimeters, whereas 21% reported not using a dosimeter at all. For high-exposure situations, the most frequently preferred protective approach was reducing exposure time (44.2%) (Supplementary Table S1).

A small subset of participants (4.3%) indicated that their hospital did not have a radiation safety committee, and 79.0% were unaware of the committee’s responsibilities. Although 82.6% stated that the committee provided training and 78.3% perceived the training as effective in improving radiation safety awareness, 27.5% reported difficulty accessing radiation safety documents. The same proportion (27.5%) perceived existing regulations as ineffective in reducing exposure for staff and patients (Supplementary Table S2).

Knowledge of regulatory dose limits was limited: only 13.8% correctly identified the 20 mSv permissible occupational dose as a 5-year average, and 32.6% correctly identified the 50 mSv annual upper limit. Most respondents (83.3%) correctly identified that a pocket dosimeter should be worn inside the lead apron; however, only 8.7% correctly identified that the radiation worker health form must be completed twice per year. All participants reported routinely asking women of childbearing age about possible pregnancy prior to ionizing-radiation examinations, and 97.8% stated that suspected cases were referred back to the requesting physician. In contrast, only 24.6% correctly identified that the optimal timing for such examinations is during menstruation and the subsequent 10 days (Supplementary Table S3).

Table 2 presents participants’ perceptions of their own knowledge regarding ionizing-radiation risks. Self-assessment scores did not differ significantly by sex, by department (radiology vs. nuclear medicine), or by work experience (≤3 years vs. >3 years). In contrast, education level was significantly associated with self-ratings (*p* = 0.001), with postgraduate participants more frequently selecting “Advanced” knowledge (15.4%) than those with undergraduate education (0.9%). Professional role was also strongly associated with self-assessment (*p* < 0.001): specialist doctors reported the highest proportion of “Advanced” knowledge (16.0%), followed by technicians (1.0%). Among the nurse and radiophysicist participants, none assessed their knowledge as “Advanced”.

Significant differences were observed across subgroups in correct responses to items assessing ionizing radiation–based methods and core radiation protection concepts. Participants with postgraduate education had a higher correct response rate for identifying ionizing-radiation methods than those with undergraduate education (100% vs. 27.5%, *p* < 0.001, Cramer’s V = 0.60). In contrast, correct identification of the ALARA principle remained high in both education groups (100% vs. 96.3%, *p* = 0.290, Cramer’s V = 0.09), whereas the correct response rate for all three basic radiation protection methods (time/duration, distance, and shielding) was higher among postgraduates than undergraduates (100% vs. 15.6%, *p* < 0.001, Cramer’s V = 0.73). Profession-related comparisons similarly revealed pronounced disparities (all *p* < 0.001; Cramer’s V range = 0.48–0.72): specialist doctors showed uniformly high correct response rates across all three outcomes (100% for ionizing-radiation methods, 100% for ALARA, and 100% for the three basic protection methods), whereas technicians showed lower correct response rates for ionizing-radiation methods (24.8%) and the three basic protection methods (14.9%), despite near-universal correct identification of ALARA (99.0%). Nurses showed intermediate correct response rates (66.7% for ionizing-radiation methods, 66.7% for ALARA, and 33.3% for the three basic protection methods). Radiophysicists were presented descriptively because of the small sample (*n* = 3). At the departmental level, nuclear medicine personnel more frequently correctly identified ionizing-radiation methods than radiology personnel (100% vs. 37.8%, *p* < 0.001, Cramer’s V = 0.34) and had a higher correct response rate for all three protection methods (81.8% vs. 29.1%, *p* < 0.001, Cramer’s V = 0.30), while correct identification of ALARA was similarly high in both departments (100% vs. 96.9%, *p* = 0.550, Cramer’s V = 0.05). No statistically significant differences were observed by sex across outcomes (all *p* > 0.05; Cramer’s V range = 0.04–0.08). With respect to work experience, correct identification of ALARA was more frequent among participants with >3 years of experience (99.1% vs. 86.4%, *p* < 0.001, Cramer’s V = 0.28), whereas the correct response rate for all three protection methods was higher among those with ≤3 years of experience (54.5% vs. 29.3%, *p* = 0.022, Cramer’s V = 0.20). Correct identification of ionizing-radiation methods did not differ significantly by work experience (59.1% vs. 39.7%, *p* = 0.104, Cramer’s V = 0.14) (Supplementary Table S4).

Knowledge of the 5-year average occupational dose limit was low overall, with only a small minority answering correctly. Profession-based differences were observed in the correct response rate for the 5-year average occupational dose limit (specialist doctor vs. technician vs. nurse: 0% vs. 12.9% vs. 33.3%; *p* < 0.001, Cramer’s V = 0.43). Radiophysicists are presented descriptively because of the very small subgroup, and all radiophysicists answered this item correctly. Department-level comparisons also showed a higher correct response rate among nuclear medicine staff than among radiology staff (63.6% vs. 9.4%, *p* < 0.001, Cramer’s V = 0.42). With respect to work experience, correct responses were more frequent among participants with ≤3 years of experience than among those with >3 years (50.0% vs. 6.9%, *p* < 0.001, Cramer’s V = 0.46). Knowledge of the annual maximum occupational dose limit also differed significantly by education, professional role, and department. Correct responses were more frequent among postgraduates than undergraduates (96.5% vs. 15.6%, *p* < 0.001, Cramer’s V = 0.70), differed across professional groups (specialist doctor vs. technician vs. nurse: 100% vs. 14.9% vs. 22.2%; *p* < 0.001, Cramer’s V = 0.73), and were more frequent among nuclear medicine staff than radiology staff (81.8% vs. 28.3%, *p* < 0.001, Cramer’s V = 0.31). No significant differences were observed by sex or work experience (Supplementary Table S5).

Correct knowledge of dosimeter placement when wearing a lead apron was more frequent among participants with >3 years of experience than among those with ≤3 years of experience (87.9% vs. 59.1%, *p* = 0.003, Cramer’s V = 0.28), while no significant differences were observed by sex, education, professional role, or department. Correct knowledge of the required frequency for completing the radiation worker health form was generally low, but differed significantly by education, professional role, department, and work experience. Correct responses were more frequent among postgraduates than undergraduates (20.7% vs. 5.5%, *p* = 0.019, Cramer’s V = 0.22), differed across professional groups (specialist doctor vs. technician vs. nurse: 8.0% vs. 3.0% vs. 44.4%; *p* < 0.001, Cramer’s V = 0.60), were more frequent among nuclear medicine staff than radiology staff (72.7% vs. 3.1%, *p* < 0.001, Cramer’s V = 0.67), and were more frequent among participants with ≤3 years of experience than among those with >3 years of experience (27.3% vs. 5.2%, *p* = 0.004, Cramer’s V = 0.29). No significant sex difference was observed (*p* = 0.490, Cramer’s V = 0.06) (Supplementary Table S6).

Correct responses regarding the recommended action when pregnancy is suspected, except in emergencies, were frequent overall. No significant subgroup differences were observed according to sex (*p* = 0.130, Cramer’s V = 0.13), education level (*p* = 0.364, Cramer’s V = 0.08), department (*p* = 0.101, Cramer’s V = 0.14), or work experience (*p* = 0.408, Cramer’s V = 0.07), although correct responses differed significantly across professional groups (specialist doctor vs. technician vs. nurse: 100% vs. 99.0% vs. 77.8%; *p* = 0.042, Cramer’s V = 0.36). In contrast, correct responses regarding the safest time to perform ionizing radiation–based procedures in women of reproductive age differed markedly across subgroups. Correct responses were more frequent among postgraduates than undergraduates (96.6% vs. 5.5%, *p* < 0.001, Cramer’s V = 0.86), differed across professional groups (specialist doctor vs. technician vs. nurse: 100% vs. 5.0% vs. 11.1%; *p* < 0.001, Cramer’s V = 0.88), and were more frequent among nuclear medicine staff than radiology staff (63.6% vs. 21.3%, *p* = 0.002, Cramer’s V = 0.27). No significant differences were observed by sex (32.2% vs. 19.0%, *p* = 0.075, Cramer’s V = 0.15) or work experience (26.7% vs. 13.6%, *p* = 0.192, Cramer’s V = 0.11). Radiophysicists were presented descriptively because of the small sample (Supplementary Table S7).

Detailed subgroup comparisons, including both statistically significant and non-significant findings with corresponding Cramer’s V values, are presented in Supplementary Tables S4–S7. Because several subgroup sizes were small or uneven, these item-level percentage differences should be interpreted descriptively rather than as definitive subgroup effects.

The total knowledge score was evaluated descriptively and by univariable linear regression according to demographic and professional characteristics (Table 3). The total score did not differ significantly by sex; male participants had a similar score to female participants (4.7 ± 1.8 vs. 4.5 ± 1.9; B = 0.26, 95% CI: −0.38 to 0.90, *p* = 0.429). In contrast, postgraduate education was associated with a higher total score than undergraduate education (7.1 ± 0.3 vs. 3.9 ± 1.5; B = 3.14, 95% CI: 2.83 to 3.46, *p* < 0.001). Professional role was also associated with the total knowledge score. Compared with specialist doctors, technicians had lower total scores (3.9 ± 1.5 vs. 7.1 ± 0.3; B = −3.19, 95% CI: −3.51 to −2.87, *p* < 0.001) and nurses also (4.8 ± 2.0 vs. 7.1 ± 0.3; B = −2.34, 95% CI: −3.78 to −0.90, *p* = 0.002). Radiophysicists had the highest descriptive score (8.7 ± 0.6), but are presented descriptively and were not included in regression analyses because of the very small subgroup. Department-level comparisons showed that nuclear medicine staff had higher total scores than radiology staff (7.1 ± 0.3 vs. 4.4 ± 1.8; B = 2.95, 95% CI: 2.27 to 3.63, *p* < 0.001). Participants with >3 years of work experience had lower total scores than those with ≤3 years of experience (4.4 ± 1.8 vs. 5.6 ± 1.8; B = −1.18, 95% CI: −2.03 to −0.33, *p* = 0.007) (Table 3).

The multivariable analysis was revised by using the total knowledge score as a continuous outcome and applying linear regression models with HC3 robust standard errors (Table 4). Because education level and professional role showed problematic collinearity when entered simultaneously, two separate adjusted models were constructed. Model 1 included sex, education level, department, and years of experience, whereas Model 2 included sex, professional role, department, and years of experience.

In Model 1, postgraduate education was associated with a higher total knowledge score than undergraduate education after adjustment for sex, department, and years of experience (B = 3.05, 95% CI: 2.61 to 3.49, *p* < 0.001). Nuclear medicine staff also had higher total scores than radiology staff (B = 1.76, 95% CI: 0.32 to 3.21, *p* = 0.017). In contrast, >3 years of work experience was associated with a lower total score compared with ≤3 years of experience (B = −1.31, 95% CI: −2.19 to −0.42, *p* = 0.004). Sex was not significantly associated with the total score (B = 0.16, 95% CI: −0.26 to 0.58, *p* = 0.445) (Table 4).

In Model 2, professional role was evaluated instead of education level, with specialist doctors as the reference category. Compared with specialist doctors, technicians had lower total knowledge scores after adjustment for sex, department, and years of experience (B = −3.09, 95% CI: −3.52 to −2.67, *p* < 0.001). Nurses also had lower total scores than specialist doctors (B = −3.84, 95% CI: −5.26 to −2.43, *p* < 0.001). The Nuclear Medicine Department remained associated with a higher total score (B = 2.33, 95% CI: 0.81 to 3.85, *p* = 0.003), while >3 years of work experience remained associated with a lower total score (B = −1.53, 95% CI: −2.40 to −0.66, *p* = 0.001). Sex was not significantly associated with the total score (B = 0.16, 95% CI: −0.27 to 0.58, *p* = 0.468) (Table 4).

## 4. Discussion

Our findings suggest that radiation safety-related self-reported practice was not fully aligned with perceived radiation-related risks. The observed discrepancy between high perceived risk and limited self-reported use of protective measures suggests a possible knowledge–practice gap, although actual clinical behavior was not directly observed. This finding suggests that familiarity with concepts such as ALARA and occupational dose limits may not be sufficient by itself to ensure consistent self-reported protective practices, particularly in busy clinical settings where workflow pressures, task prioritization, and local departmental routines may influence day-to-day behavior. Similar gaps between knowledge and self-reported protective practices have been reported in previous hospital-based studies [14,15,17]. Rather than representing a major conceptual advance in radiation safety research, the present study should be interpreted as a local institutional assessment of radiation safety knowledge, perceptions, and self-reported practices within the same healthcare setting. Its main contribution lies in comparing two related but operationally distinct radiation-use environments, radiology and nuclear medicine, and identifying role- and department-specific gaps that may inform targeted institutional training, monitoring, and procedural visibility. This approach may help identify department- and role-specific differences that are less apparent when only one professional group or one clinical setting is examined. Accordingly, our findings may help identify areas where future targeted radiation safety programs could be considered, including role-specific training, department-adapted monitoring, and reinforcement of self-reported routine protective practices. Taken together, these findings suggest that education, clear institutional procedures, accessible protective equipment, and regular monitoring and feedback may support radiation protection practices.

In our sample, infrequent PPE use and irregular dosimeter use were commonly reported, which broadly resonates with previous hospital-level studies. A previous study reported that some HCWs rarely implemented radiation precautions beyond basic behaviors such as standing behind shielding or leaving the room, and that protective items such as lead aprons and thyroid collars were not used routinely [17]. In another study, inadequate supply of some PPE, especially lead glasses and gloves, the availability or location of barriers, and physical discomfort were frequently reported as barriers to PPE use, whereas forgetfulness was the main rationale for not wearing a dosimeter [28]. Similarly, data from Main Assiut University showed substantial deficits, with a majority of HCWs demonstrating poor knowledge of radiation hazards and more than two-thirds showing poor practical application score [29].

However, not all studies have reported the same pattern: some have found relatively adequate radiation safety knowledge among HCWs [30,31]. These discrepancies may be partly explained by differences in study population composition, including professional role, unit, and exposure profile. Beyond knowledge, practical barriers such as heavy or uncomfortable lead aprons, time constraints, and normalization of risk may contribute to lower self-reported use of protective measures. Current multicenter evidence still suggests that radiation protection awareness among medical personnel is moderate to poor on average [15]. Altogether, this evidence supports the need to interpret radiation safety knowledge and self-reported practices in relation to professional role, departmental context, and local workflow.

This study found that staff with postgraduate education had higher radiation safety knowledge scores and higher correct-response rates for some knowledge items than those with undergraduate education. In a study among Egyptian healthcare personnel, a positive association was also found between educational status and radiation safety awareness scores [17]. Evidence from other contexts also suggests that knowledge and practice patterns may differ according to job function. An Iranian study reported that nurses working in interventional radiology had lower radiation protection knowledge and practice scores than doctors, even in the same work environment [32]. Similarly, a medical college-based study in Nepal indicated that radiology staff who were undergraduate degree holders demonstrated significantly lower radiation protection knowledge compared to employees with postgraduate degrees [33]. Although the extent and format of previous training were not directly assessed, postgraduate participants may have had more structured exposure to radiation physics and radiation protection, which may partly explain their higher scores on knowledge-based items. However, differences by profession in our study were not homogeneous across all domains, suggesting that role-specific workflows and training emphasis may be associated with performance across topic-related domains. Interestingly, technicians in our sample had near-universal recognition of the ALARA principle, but lower correct response rates on items requiring identification of ionizing radiation–based modalities, suggesting a possible distinction between familiarity with general principles and detailed modality-specific knowledge. These results are consistent with previous studies showing that radiation-related knowledge is generally higher among specialists and staff who are more frequently involved in ionizing radiation procedures and protection practices [15,34]. Borgen et al. noted that radiologists and radiographers showed a marked relative performance advantage over other referring physicians in radiation dose and protection knowledge [34].

A striking finding was that nuclear medicine staff tended to achieve higher correct response rates than radiology staff on several radiation safety items, particularly those related to recognizing ionizing-radiation methods and selected protection strategies. While some department-level differences were pronounced, not all comparisons reached statistical significance, and the relatively small nuclear medicine subgroup warrants cautious interpretation of the results. Future studies should directly evaluate departmental safety culture, workflow characteristics, and institutional training practices to determine whether these factors explain the observed differences.

These findings are in line with the wider literature indicating that radiation protection knowledge and self-reported practices vary across institutions and departments. A Jordanian study indicated that radiology personnel achieved the highest radiation protection scores among hospital departments [15], whereas a separate study reported that a substantial proportion of non-radiologists may still be unable to distinguish between ionizing and non-ionizing imaging modalities [30]. Similarly, a multicenter survey from Pakistan found that radiation workers across radiology, nuclear medicine, and radiotherapy had limited awareness of protection-related issues on average, with only a subset correctly answering questions such as recognition of warning symbols and understanding of annual dose limits [35]. Although these factors were not directly examined in our study, one possible explanation is that differences in routine protocols, supervision intensity, and training emphasis between units may affect both radiation safety knowledge and self-reported practice. In many nuclear medicine settings, closer involvement of radiation safety officers and routine monitoring practices (e.g., contamination checks and badge compliance monitoring) may provide stronger reinforcement, whereas radiology workflows may be more variable and distributed across procedures and teams than in nuclear medicine. Overall, these findings suggest the potential value of cross-departmental training and standardization of core radiation safety protocols to reduce variability and support high-quality radiation protection practices across units [30,35,36].

One of the more nuanced findings of this study is the mixed association between work experience and radiation safety knowledge and self-reported practices. In our study, more experienced staff (>3 years) showed higher correct response rates on certain knowledge items (e.g., correct dosimeter placement when wearing a lead apron) and showed higher knowledge of core principles such as ALARA, whereas early career staff (≤3 years) more often answered correctly on selected rule-based or procedural knowledge items, including knowledge of occupational dose-limit concepts and the required frequency for completing the radiation worker health form. Several possible explanations may account for this pattern, although they were not directly tested in this cross-sectional study. First, knowledge decay may be one possible explanation when concepts are not revisited regularly, particularly if formal instruction is concentrated during initial onboarding. Second, reduced exposure to structured continuing education after initial training may be another possible explanation, and day-to-day workflow demands may shift attention toward practical task completion rather than revisiting theoretical principles. Third, differences in unit-specific safety culture (e.g., routine audits, feedback, and refresher training) may differentially reinforce knowledge over time. A further possibility is a cohort effect, whereby newer staff benefit from more recent curricula or guideline updates. In the adjusted linear regression models, >3 years of work experience was associated with a lower total knowledge score. However, this finding should be interpreted cautiously because of the cross-sectional design, the unbalanced subgroup structure, and the possibility of residual confounding; causality cannot be inferred, and longitudinal studies are warranted to clarify these possible explanations. In addition, education level and professional role were evaluated in separate adjusted models because of problematic multicollinearity; therefore, the experience-related finding should not be interpreted as a causal or independent determinant. This lack of a linear experience–knowledge relationship has previously been observed. Fataftah et al. reported only a weak, non-significant correlation between years of service and radiation protection knowledge, suggesting that “awareness of ionizing radiation is not automatically acquired over time” [15]. Professional experience has, however, not been found to have a significant impact on radiation safety awareness in some studies [30,37,38,39], whereas a few others have reported modest positive effects associated with job-related cumulative learning [17,40]. Some evidence also suggests that junior staff may outperform more experienced colleagues on knowledge tests, possibly because of more recent theoretical training [41,42]. A systematic review pointed out that regardless of age or seniority, many participants demonstrated only average knowledge, and that “experience alone does not guarantee up-to-date safety understanding” [14]. Together, these findings suggest the potential value of continuous professional development: rather than presuming that expertise increases naturally with tenure, institutions may consider ongoing refresher training in updated radiation safety protocols applicable to all staff.

### 4.1. Clinical Implications

The following implications are presented as practical suggestions informed by our findings and should be adapted to local regulations and institutional resources. Taken together, our results suggest several pragmatic areas for institutional improvement, although these recommendations should be interpreted as practice-oriented suggestions rather than causal conclusions derived directly from the study design. First, role-based targeted education may help address gaps in groups with lower knowledge scores. Targeted learning opportunities for technicians and nurses in imaging-related departments may include specialized workshops on basic radiation concepts, dose optimization, and the appropriate selection and use of PPE. However, previous studies suggest that such interventions may improve knowledge and adherence to protective behaviors [15,37]. Consistent with this rationale, our findings support considering structured and periodic radiation safety training for all personnel exposed to ionizing radiation, rather than relying only on optional or ad hoc training. Second, institutional policy and leadership support may help strengthen the translation of knowledge into routine safety-related practice. Institutions should ensure that protective equipment, including lead aprons, thyroid shields, dosimeters, and shielding barriers, is available, functional, and routinely maintained. Clear policies may also create common expectations for use (e.g., dosimeters will be used in a consistent way) and provide accountability mechanisms (such as checks for periodic badge/PPE adherence) that are commonly used in regulated workplaces [17,37,43]. Practical considerations may include annual refresher courses, the development of new e-learning materials and simulation exercises for radiological emergencies or high-exposure circumstances Finally, routine reinforcement of radiation protection practices may be important. In addition to established guidance and instruction, staff should be supported in internalizing ALARA (“as low as reasonably achievable”) and recognizing that minimizing exposure is a team task. Visible reminders by radiation safety officers or designated unit staff may help reinforce appropriate dosimeter placement, regular PPE use, appropriate shielding, and adherence to dose-reduction protocols during daily routines. Collectively, these measures may help sustain awareness and support more consistent self-reported protective practices across roles, departments, and experience levels.

### 4.2. Limitations

There are several limitations when interpreting our results. First, the study population was drawn from staff in radiology and nuclear medicine in a single setting, which may compromise the external validity of the results. Radiation safety practices and training frameworks are institutionally and nationally specific, so the results may not be completely applicable to other healthcare settings. Although the response rate was acceptable, non-response bias cannot be fully excluded because 21 eligible healthcare workers did not participate. Since participation was voluntary and no individual-level data were collected from non-participants, a formal participant–non-participant comparison could not be performed. Non-participation may have been related to workload, professional role, previous training, or interest in radiation safety. In addition, subgroup sizes were markedly unbalanced across department and professional-role categories. Accordingly, subgroup comparisons should be interpreted as exploratory descriptive associations rather than evidence of independent group effects, particularly for small subgroups such as nuclear medicine staff, nurses, and radiophysicists. The sample size calculation was based on a conservative single-proportion framework related to the overall knowledge outcome and was not designed as a formal power calculation for multiple item-level comparisons or adjusted regression models. Therefore, the calculated sample size should not be interpreted as providing adequate statistical power for all subgroup comparisons or for the multivariable models. Although the total knowledge score was analyzed as a continuous outcome using HC3 robust standard errors, the unbalanced subgroup structure may still have reduced statistical precision and may have inflated observed percentage contrasts in either direction. In addition, education level and professional role were not included simultaneously in the same adjusted model because of problematic multicollinearity; therefore, these constructs were evaluated in separate models. Second, because of the cross-sectional design, the study reflects perceptions and self-reported practices at a single point in time and does not allow causal inference. Third, knowledge was assessed using predefined correct responses, whereas awareness, perceptions, and practices were assessed using participant-reported questionnaire responses; practice-related behaviors were not directly observed. These assessment approaches may have introduced recall bias, interviewer influence, and social-desirability bias. This is particularly relevant for safety-related behaviors, because participants may have felt pressure to provide more acceptable answers during face-to-face interviews. Respondents may have overreported adherence to protective behaviors or misremembered factual information, and no direct observation, audit, or objective testing was performed to validate these responses. Therefore, actual clinical behavior—particularly regarding routine PPE use and dosimeter wearing—may have differed from the reported patterns. Finally, although the questionnaire covered broad domains of radiation safety, it may not have captured all factors associated with self-reported protective practice. Although the questionnaire was developed using radiation protection guidance, adapted from previous hospital-based surveys, reviewed by domain experts, and pilot tested before use, formal psychometric validation across the knowledge, awareness, perception, and self-reported practice domains was beyond the scope of this study. In addition, the all-or-none scoring approach used for selected knowledge items may have underestimated partial knowledge, particularly for multi-component and multi-response items. Cronbach’s alpha for the knowledge-based items indicated only minimally acceptable internal consistency, and this should be interpreted cautiously because the knowledge items covered heterogeneous content areas and varied in difficulty. Factors such as workload, time pressure, institutional safety climate, routine audits, enforcement of dosimeter policies, and the availability or condition of protective equipment may also influence day-to-day practice. Future studies should use larger, preferably multicenter samples, apply fully validated instruments where feasible, consider graded scoring systems for partial knowledge, and use robust adjusted modeling strategies, ideally complemented by observational or mixed-method approaches.

## 5. Conclusions

This study identified gaps and differences in radiation safety knowledge, awareness, and self-reported practices among HCWs in radiology and nuclear medicine settings. Importantly, the findings suggest that knowledge of basic principles was not consistently aligned with self-reported protective practices in routine clinical workflows. Therefore, targeted, role-specific training together with clear institutional procedures and regular monitoring may help support more consistent radiation safety practices and improve the visibility and sustainability of institutional radiation protection procedures.

## Figures and Tables

**Table 1:** Demographic characteristics of the participants.

Variables	Total Population *n* = 138
Age, years	39.4 ± 8.2
Female, *n* (%)	79 (57.2)
Education, *n* (%)	
Undergraduate	109 (79.0)
Postgraduate	29 (21.0)
Professional, *n* (%)	
Specialist doctor	25 (18.1)
Technician	101 (73.2)
Nurse	9 (6.5)
Radiophysicist	3 (2.2)
Department, *n* (%)	
Radiology	127 (92.0)
Nuclear Medicine	11 (8.0)
Experience in the field of work, *n* (%)	
≤3 years	22 (15.9)
>3 years	116 (84.1)

Data are presented as means ± standard deviation or frequency (column percentage).

**Table 2:** Participants’ self-assessment of knowledge concerning ionizing-radiation risks.

Variables	*n* ^b^	Advanced	Sufficient	Insufficient	*p*
Sex					
Female	79	2 (2.5)	72 (91.1)	5 (6.3)	0.318
Male	59	3 (5.1)	55 (93.2)	1 (1.7)	
Education					
Undergraduate	109	1 (0.9)	103 (94.5)	5 (4.6)	0.001
Postgraduate	29	**4 (15.4)**	**21 (80.8)**	1 (3.8)	
Professional					
Specialist doctor	25	**4 (16.0)**	21 (84.0)	0	<0.001
Technician	101	1 (1.0)	97 (96.0)	3 (3.0)	
Nurse	9	0	**6 (66.7)**	**3 (33.3)**	
Radiophysicist ^a^	3	0	3(100)	0	
Department					
Radiology	127	5 (3.9)	117 (92.1)	5 (3.9)	0.591
Nuclear medicine	11	0	10 (90.9)	1 (9.1)	
Experience in the field of work					
≤3 years	22	1 (4.5)	18 (81.8)	3 (13.6)	0.217
>3 years	116	4 (3.4)	109 (94.0)	3 (2.6)	

Data are presented as frequency (row percentage). Significant Bonferroni-adjusted post hoc pairwise differences are indicated in bold. ^a^ Radiophysicists (*n* = 3) were excluded from statistical analyses due to the small sample and are presented descriptively. ^b^ Denominator values.

**Table 3:** Distribution of the total knowledge score and univariable linear regression results according to participant characteristics.

Variables	Total Knowledge Score	Univariable Regression			VIF
		B ± SE	95% CI	*p*	
Sex, *n* (%)					1.06
Female	4.5 ± 1.9	ref			
Male	4.7 ± 1.8	0.26 ± 0.22	−0.38 to 0.90	0.429	
Education, *n* (%)					10.36
Undergraduate	3.9 ± 1.5	ref			
Postgraduate	7.1 ± 0.3	3.14 ± 0.16	−2.83 to 3.46	<0.001	
Professional, *n* (%)					10.57
Specialist doctor	7.1 ± 0.3	ref			
Technician	3.9 ± 1.5	−3.19 ± 0.16	−3.51 to −2.87	<0.001	
Nurse	4.8 ± 2.0	−2.34 ± 0.73	−3.78 to −0.90	0.002	
Radiophysicist ^a^	8.7 ± 0.6	not included			
Department, *n* (%)					1.15
Radiology	4.4 ± 1.8	ref			
Nuclear medicine	7.1 ± 0.3	2.95 ± 0.34	2.27 to 3.63	<0.001	
Experience in the field of work, *n* (%)					1.10
≤3 years	5.6 ± 1.8	ref			
>3 years	4.4 ± 1.8	−1.18 ± 0.43	−2.03 to −0.33	0.007	

B, regression coefficient; SE, standard error; CI, confidence interval; VIF, variance inflation factor. Reference categories were female sex, undergraduate education, specialist doctor, radiology, and ≤3 years of experience. ^a^ Radiophysicists are presented descriptively and were not included in regression analyses.

**Table 4:** Multivariable linear regression models with HC3 robust standard errors for factors associated with the total knowledge score.

Variables	Model I Regression			VIF	Model II Regression			VIF
	B ± SE	95% CI	*p*		B ± SE	95% CI	*p*	
Sex, *n* (%)				1.06				1.06
Female								
Male	0.16 ± 0.21	−0.26 to 0.58	0.445		0.16 ± 0.22	−0.27 to 0.58	0.468	
Education, *n* (%)				1.08				1.10
Undergraduate	ref				not included			
Postgraduate	3.05 ± 0.22	2.61 to 3.49	<0.001					
Professional, *n* (%)								1.10
Specialist doctor	not included				ref			
Technician					−3.09 ± 0.22	−3.52 to −2.67	<0.001	
Nurse					−3.84 ± 0.72	−5.26 to −2.43	<0.001	
Department, *n* (%)				1.10				1.13
Radiology	ref				ref			
Nuclear medicine	1.76 ± 0.73	0.32 to 3.21	0.017		2.33 ± 0.77	0.81 to 3.85	0.003	
Experience in the field of work, *n* (%)				1.09				1.08
≤3 years	ref				ref			
>3 years	−1.31 ± 0.45	−2.19 to −0.42	0.004		−1.53 ± 0.44	−2.40 to −0.66	0.001	

B, regression coefficient; SE, standard error; CI, confidence interval; VIF, variance inflation factor. HC3 robust standard errors were used. Model 1 included sex, education level, department, and years of experience. Model 2 included sex, professional role, department, and years of experience. Professional role was entered using specialist doctors as the reference category. Radiophysicists were excluded from regression models because of the very small subgroup and are presented descriptively. VIF values were obtained from the corresponding ordinary least squares models using the same covariate sets.

## Data Availability

Anonymized participant data, survey data, and the full questionnaire will be provided upon reasonable request for research purposes. Requests should be sent to the corresponding author at eda.pepele@ozal.edu.tr. Access will be granted in accordance with the ethics approval of the study and applicable data protection regulations and may require a data usage agreement. Data that could allow reidentification will not be shared.

## Supplementary Materials

The following supporting information can be downloaded at https://www.mdpi.com/article/10.3390/healthcare14152370/s1. Table S1. Distribution of perceptions and self-reported practices related to radiation protection; Table S2: Distribution of responses regarding the radiation safety committee and institutional radiation safety procedures; Table S3: Distribution of answers to questions measuring the level of knowledge about radiation safety and protection; Table S4: Knowledge of ionizing radiation–based modalities and radiation protection principles; Table S5: Knowledge of occupational dose limits; Table S6: Applied radiation safety knowledge and routine procedural practices; Table S7: Knowledge of radiation safety procedures for women of childbearing age.
