# Supplementary material for: Radiation Safety Knowledge, Perceptions, and Self-Reported Practices Among Healthcare Workers in Radiology and Nuclear Medicine: A Cross-Sectional Survey
Source: Healthcare (Basel). 2026 Aug 3;14(15):2370. doi: 10.3390/healthcare14152370 (PMC13464716; doi:10.3390/healthcare14152370)
Supplement: Supplementary file 1 [file healthcare-14-02370-s001.zip › healthcare-4176376-supplementary.pdf]

Table S1. Distribution of perceptions and self-reported practices related to radiation protection.

Questionnaire item		n	% (95% CI)	Denominator
How did you acquire knowledge about radiation protection? *	During my education	131	94.9 (89.9–97.5)	138
	Through training provided by the hospital where I work	86	62.3 (54.0–70.0)	138
	Through training provided by the device company	7	5.1 (2.5–10.1)	138
	Through courses provided by NDK	8	5.8 (3.0–11.0)	138
	Through the internet and media	9	6.5 (3.5–11.9)	138
	Through my colleagues	8	5.8 (3.0–11.0)	138
Do you think your risk of radiation exposure is higher than in other units?	Yes	133	96.4 (91.8–98.4)	138
	No	5	3.6 (1.6–8.2)	
Which PPE do you use to protect against radiation risks?*	Lead glasses	5	3.6 (1.6–8.2)	138
	Lead gloves	3	2.2 (0.7–6.2)	138
	Gonad protector	4	2.9 (1.1–7.2)	138
	Lead screen	14	10.1 (6.1–16.3)	138
	Lead apron	19	13.8 (9.0–20.5)	138
	Thyroid protector	14	10.1 (6.1–16.3)	138
	Leaded glass	12	8.7 (5.0–14.6)	138
	None	85	61.6 (53.3–69.3)	138
Which dosimeter do you use regularly?	Pocket dosimeter	104	75.4 (67.6–81.8)	138
	Pocket dosimeter and wrist dosimeter	5	3.6 (1.6–8.2)	
	I do not use a dosimeter	29	21.0 (15.0–28.6)	
If you are not using a dosimeter, why?	I forget	3	10.3 (3.6–26.4)	29[ǂ]
	I don't think the dosimeter is measuring correctly	4	13.8 (5.5–30.6)	
	Non-response	22	75.9 (57.9–87.8)	
What measures do you take to reduce exposure in case of high exposure?*	Exposure time is reduced	61	44.2 (36.2–52.5)	138
	I use PPE	47	34.1 (26.7–42.3)	138
	I expect the radiation protection officer to intervene in the incident and precautions to be taken to prevent recurrence of overdose.	49	35.5 (28.0–43.8)	138
	I have no idea	5	3.6 (1.6–8.2)	138

For the multi-response item (*), participants could select more than one option. Percentages were calculated as (number selecting the option / total respondents, n = 138) × 100; therefore, totals may exceed 100%. For single-response items, percentages were calculated over n = 138. [ǂ] The assessment was conducted among participants who reported not using a dosimeter. For the PPE item, participants could select more than one PPE option; however, 'None' was treated as a mutually exclusive response and indicated that none of the listed PPE items were used.

Table S2. Distribution of answers to questions about the radiation safety committee.

Questionnaire item		n	% (95% CI)	Denominator
Is there a radiation safety committee in the hospital?	Yes	132	95.7 (90.8–98.0)	138
	No	6	4.3 (2.0–9.2)	
Do you know the responsibilities of the radiation safety committee?	Yes	29	21.0 (15.0–28.6)	138
	No	109	79.0 (71.4–85.0)	
Is radiation safety training provided in the hospital?	Yes	114	82.6 (75.4–88.0)	138
	No	24	17.4 (12.0–24.6)	
Do you think the training is effective in improving radiation safety awareness?	Yes	108	78.3 (70.7–84.3)	138
	No	30	21.7 (15.7–29.3)	
Is there a radiation safety procedures document in the hospital?	Yes, it is available in our clinic	25	18.1 (12.6–25.4)	138
	Yes, I can access it through our hospital's website	75	54.3 (46.0–62.4)	
	No, there is no such document, or I cannot access it.	38	27.5 (20.8–35.5)	
Do you think radiation safety regulations have reduced radiation exposure among staff and patients?	Yes	100	72.5 (64.5–79.2)	138
	No	38	27.5 (20.8–35.5)	
Do you inform patients about radiation risks?*	Using the patient consent form	57	41.3 (33.4–49.6)	138
	Only for patients under 18 years of age	78	56.5 (48.2–64.5)	
	Only for CT imaging	72	52.2 (43.9–60.3)	

*For the multi-response item on informing patients about radiation risk, participants could select more than one option. Therefore, percentages represent the proportion of respondents selecting each option and should not be interpreted as mutually exclusive categories; totals may exceed 100%. Percentages were calculated as (number selecting the option / total respondents, n = 138) × 100; therefore, totals may exceed 100%. For single-response items, percentages were calculated over n = 138.

Table S3. Distribution of answers to questions measuring the level of knowledge about radiation safety and protection.

Questionnaire item		n	% (95% CI)	Denominator
What level of knowledge do you think you have about the risks of ionizing radiation?	Advanced	5	3.6 (1.6–8.2)	138
	Sufficient	127	92.0 (86.3–95.5)	
	Insufficient	6	4.3 (2.0–9.2)	
Which modalities use ionizing radiation?*	**Chest X-ray**	50	36.2 (28.7–44.5)	138
	**Mammography**	48	34.8 (27.3–43.0)	138
	**PET**	50	36.2 (28.7–44.5)	138
	MRI	2	1.4 (0.4–5.1)	138
	**Fluoroscopy**	40	29.0 (22.1–37.0)	138
	**Interventional radiology applications**	44	31.9 (24.7–40.1)	138
	**Cardiac angiography studies**	43	31.2 (24.0–39.3)	138
	**Radiation therapy (radiotherapy)**	43	31.2 (24.0–39.3)	138
	Ultrasound	77	55.8 (47.5–63.8)	138
	**Bone densitometry**	124	89.9 (83.7–93.9)	138
	**CT**	61	44.2 (36.2–52.5)	138
	**Scintigraphy imaging**	49	35.5 (28.0–43.8)	138
	**I-131 treatment**	40	29.0 (22.1–37.0)	138
Which option corresponds to the ALARA principle?	**Lowest possible radiation dose**	134	97.1 (92.8–98.9)	138
	I don't know	4	2.9 (1.1–7.2)	
Which option includes the basic methods of radiation protection?	**Time, distance, and shielding**	46	33.3 (26.0–41.6)	138
	Incorrect or missing answer	92	66.7 (58.4–74.0)	
What is the 5-year average occupational dose limit for radiation workers?	1 mSv	4	2.9 (1.1–7.2)	138
	5 mSv	17	12.3 (7.8–18.8)	
	**20 mSv**	19	13.8 (9.0–20.5)	
	50 mSv	7	5.1 (2.5–10.1)	
	I don't know	91	65.9 (57.7–73.3)	
What is the annual maximum occupational dose limit for radiation workers?	1 mSv	1	0.7 (0.1–4.0)	138
	5 mSv	86	62.3 (54.0–70.0)	
	20 mSv	2	1.4 (0.4–5.1)	
	**50 mSv**	45	32.6 (25.4–40.8)	
	I don't know	4	2.9 (1.1–7.2)	
Where should a pocket dosimeter be placed when using a lead apron?	On your apron	23	16.7 (11.4–23.8)	138
	**Under the apron**	115	83.3 (76.2–88.6)	
Required frequency for completing the radiation worker health form	**Once every 6 months**	12	8.7 (5.0–14.6)	138
	Once every 2 years	26	18.8 (13.2–26.2)	
	I don't know	100	72.5 (64.5–79.2)	
Pregnancy questioning before examinations using ionizing radiation in women of childbearing age	**Yes**	138	100.0 (97.3–100.0)	138
What do you do if pregnancy is suspected in women of childbearing age (except in emergencies)?	**Referred to the physician requesting the examination**	135	97.8 (93.8–99.3)	138
	The individual's declaration is taken as the basis	2	1.4 (0.4–5.1)	
	The statement of the individual's family is taken as the basis	1	0.7 (0.1–4.0)	
What is the safest time to perform techniques using ionizing radiation in women of childbearing age?	1 week before menstruation	10	7.2 (4.0–12.8)	138
	**During menstruation and the following 10 days**	34	24.6 (18.2–32.4)	
	2 weeks before menstruation	83	60.1 (51.8–67.9)	
	2 weeks after menstruation	11	8.0 (4.5–13.7)	

*For multi-response items, participants could select more than one option. Percentages were calculated as (number selecting the option / total respondents, n = 138) × 100; therefore, totals may exceed 100% and response options should not be interpreted as mutually exclusive categories. For single-response items, percentages were calculated over n = 138. Bold characters indicate the correct answer.

Table S4. Knowledge of ionizing radiation-based modalities and radiation protection principles.

Variables	n[b]	Knowledge of methods using ionizing radiation				Knowledge of the ALARA principle				Knowledge of all three basic methods of radiation protection (duration, distance, shielding)			
		Correct answer	Non-correct answer	p	Cramer's V	Correct answer	Non-correct answer	p	Cramer's V	Correct answer	Non-correct answer	p	Cramer's V
Gender, n (%)				0.334	0.08			0.466	0.06			0.626	0.04
Female	79	31 (39.2)	48 (60.8)			76 (96.2)	3 (3.8)			25 (31.6)	54 (68.4)		
Male	59	28 (47.5)	31 (52.5)			58 (98.3)	1 (1.7)			21 (35.6)	38 (64.4)		
Education, n (%)				<0.001	0.60			0.290	0.09			<0.001	0.73
Undergraduate	109	30 (27.5)	79 (72.5)			105 (96.3)	4 (3.7)			17 (15.6)	92 (84.4)		
Postgraduate	29	29 (100)	0			29 (100)	0			29 (100)	0		
Professional role, n (%)				<0.001	0.62			<0.001	0.48			<0.001	0.72
Specialist doctor	25	**25 (100)**	**0**			25 (100)	0			**25 (100)**	**0**		
Technician	101	25 (24.8)	76 (75.2)			100 (99.0)	1 (1.0)			15 (14.9)	86 (85.1)		
Nurse	9	6 (66.7)	3 (33.3)			**6 (66.7)**	**3 (33.3)**			3 (33.3)	6 (66.7)		
Radiophysicist[a]	3	3 (100)	0			3 (100)	0			3 (100)	0		
Department, n (%)				<0.001	0.341			0.550	0.05			<0.001	0.30
Radiology	127	48 (37.8)	79 (100.0)			123 (96.9)	4 (3.1)			37 (29.1)	90 (70.9)		
Nuclear Medicine	11	11 (100)	0			11 (100)	0			9 (81.8)	2 (18.2)		
Experience in the field of work, n (%)				0.104	0.144			<0.001	0.28			0.022	0.20
≤ 3 years	22	13 (59.1)	9 (40.9)			19 (86.4)	3 (13.6)			12 (54.5)	10 (45.5)		
> 3 years	116	46 (39.7)	70 (60.3)			115 (99.1)	1 (0.9)			34 (29.3)	82 (70.7)		

Data are presented as frequency (row percentage). For professional-role comparisons, significant Bonferroni-adjusted post hoc pairwise differences are indicated in bold. [a]Radiophysicists (n = 3) were excluded from statistical analyses due to the small sample size and were presented descriptively. [b] indicates the denominator values. Correct answers were defined according to established radiation protection standards (time–distance–shielding; ALARA; and identification of ionizing modalities).

Table S5. Knowledge of occupational dose limits.

Variables	n[b]	Knowledge of the 5-year average dose allowed				Knowledge of the annual maximum dose allowed			
		Correct answer	Non-correct answer	p	Cramer's V	Correct answer	Non-correct answer	p	Cramer's V
Gender, n (%)				0.575	0.05			0.780	0.02
Female	79	12 (15.2)	67 (84.8)			25 (31.6)	54 (68.4)		
Male	59	7 (11.9)	52 (88.1)			20 (33.9)	39 (66.1)		
Education, n (%)				0.999	0.01			<0.001	0.70
Undergraduate	109	15 (13.8)	94 (86.2)			17 (15.6)	92 (84.4)		
Postgraduate	29	4 (13.8)	25 (86.2)			28 (96.5)	1 (3.5)		
Professional role, n (%)				<0.001				<0.001	0.73
Specialist doctor	25	0	25 (100)		0.43	**25 (100)**	**0**		
Technician	101	13 (12.9)	88 (87.1)			15 (14.9)	86 (85.1)		
Nurse	9	**3 (33.3)**	**6 (66.7)**			2 (22.2)	7 (77.8)		
Radiophysicist[a]	3	3 (100)	0			3 (100)	0		
Department, n (%)				<0.001	0.42			<0.001	0.31
Radiology	127	12 (9.4)	115 (90.6)			36 (28.3)	91 (71.7)		
Nuclear Medicine	11	7 (63.6)	4 (36.4)			9 (81.8)	2 (18.2)		
Experience in the field of work, n (%)				<0.001	0.46			0.365	0.12
≤ 3 years	22	11 (50)	11 (50)			10 (45.5)	12 (54.5)		
> 3 years	116	8 (6.9)	108 (93.1)			35 (30.2)	81 (69.8)		

Data are presented as frequency (row percentage). For professional-role comparisons, significant Bonferroni-adjusted post hoc pairwise differences are indicated in bold. [a] Radiophysicists (n = 3) were excluded from statistical analyses because of the small sample size and are presented descriptively. [b] Indicates denominator values. Correct answers were defined according to established radiation protection standards, including time–distance–shielding, ALARA, and identification of ionizing radiation-based modalities.

Table S6. Applied radiation safety knowledge and routine procedural practices.

Variables	n[b]	Correct Placement of Personal Dosimeter with Lead Apron				Required Frequency for Radiation Worker Health Form			
		Correct answer	Non-correct answer	p	Cramer's V	Correct answer	Non-correct answer	p	Cramer's V
Gender, n (%)				0.397	0.07			0.490	0.06
Female	79	64 (81.0)	15 (19.0)			8 (10.1)	71 (89.9)		
Male	59	51 (86.4)	8 (13.6)			4 (6.8)	55 (93.2)		
Education, n (%)				0.097	0.18			0.019	0.22
Undergraduate	109	87 (79.8)	22 (20.2)			6 (5.5)	103 (94.5)		
Postgraduate	29	28 (96.5)	1 (3.5)			6 (20.7)	23 (79.3)		
Professional role, n (%)				0.221	0.17			<0.001	0.60
Specialist doctor	25	24 (96.0)	1 (4.0)			2 (8.0)	23 (92.0)		
Technician	101	81 (80.2)	20 (19.8)			3 (3.0)	98 (97.0)		
Nurse	9	7 (77.8)	2 (22.2)			**4 (44.4)**	**5 (55.6)**		
Radiophysicist[a]	3	3 (100)	0			3 (100)	0		
Department, n (%)				0.482	0.06			<0.001	0.67
Radiology	127	105 (82.7)	22 (17.3)			4 (3.1)	123 (96.9)		
Nuclear Medicine	11	10 (90.9)	1 (9.1)			8 (72.7)	3 (27.3)		
Experience in the field of work, n (%)				0.003	0.28			0.004	0.29
≤ 3 years	22	13 (59.1)	9 (40.9)			6 (27.3)	16 (72.7)		
> 3 years	116	102 (87.9)	14 (12.1)			6 (5.2)	110 (94.8)		

Data are presented as frequency (row percentage). For professional-role comparisons, significant Bonferroni-adjusted post hoc pairwise differences are indicated in bold.
[a]Radiophysicists (n = 3) were excluded from statistical analyses due to the small sample size and were presented descriptively. Correct placement and required frequency were defined according to institutional radiation safety procedures.

Table S7. Knowledge of radiation safety procedures for women of childbearing age.

Variables	n[b]	What do you do if pregnancy is suspected in women of childbearing age (except in emergencies)?				What is the safest time to perform techniques using ionizing radiation in women of childbearing age?			
		Correct answer	Non-correct answer	p	Cramer's V	Correct answer	Non-correct answer	p	Cramer's V
Gender, n (%)				0.130	0.13			0.075	0.15
Female	79	76 (96.2)	3 (3.8)			15 (19.0)	64 (81.0)		
Male	59	59 (100)	0			19 (32.2)	40 (67.8)		
Education, n (%)				0.364	0.08			<0.001	0.86
Undergraduate	109	106 (97.2)	3 (2.8)			6 (5.5)	103 (94.5)		
Postgraduate	29	29 (100)	0			28 (96.6)	1 (3.4)		
Professional role, n (%)				0.042	0.36			<0.001	0.88
Specialist doctor	25	25 (100)	0			**25 (100)**	**0**		
Technician	101	100 (99.0)	1 (1.0)			5 (5.0)	96 (95.0)		
Nurse	9	**7 (77.8)**	**2 (22.2)**			1 (11.1)	8 (88.9)		
Radiophysicist[a]	3	3 (100)	0			3 (100)	0		
Department, n (%)				0.101	0.14			0.002	0.27
Radiology	127	125 (98.4)	2 (1.6)			27 (21.3)	100 (78.7)		
Nuclear Medicine	11	10 (90.9)	1 (9.1)			7 (63.6)	4 (36.4)		
Experience in the field of work, n (%)				0.408	0.07			0.192	0.11
≤ 3 years	22	21 (95.5)	1 (4.5)			3 (13.6)	19 (86.4)		
> 3 years	116	114 (98.3)	2 (1.7)			31 (26.7)	85 (73.7)		

Data are presented as frequency (row percentage). For professional-role comparisons, significant Bonferroni-adjusted post hoc pairwise differences are indicated in bold. [a]Radiophysicists (n = 3) were excluded from statistical analyses due to the small sample size and were presented descriptively. Correct responses for pregnancy-related items were defined according to standard radiation protection recommendations for women of childbearing age. [b]Indicates denominator values.
